# Mimicking Polycystic Liver Disease: A Case Report of Innumerable Cystic Hepatic Metastases From a Pancreatic Neuroendocrine Tumour and Diagnostic Role of Endoscopic Ultrasound

**DOI:** 10.7759/cureus.115762

**Published:** 2026-09-04

**Authors:** Qais Payab, Rida Abbasi, Mohammed Korani, Ajith Siriwardena, Venkata PK Lekharaju, Daniyal Baig, Javaid Iqbal, Sajjad Mahmood

**Affiliations:** 1 Gastroenterology and Hepatology, Wythenshawe Hospital, Manchester University NHS Foundation Trust, Manchester, GBR; 2 Hepato-Pancreato-Biliary Surgery, Manchester Royal Infirmary, Manchester University NHS Foundation Trust, Manchester, GBR

**Keywords:** chronic lymphoid leukemia, endoscopic ultrasound (eus), fine needle aspiration (fna), hepatic metastases, liver cysts, main pancreatic duct dilatation, nonfunctional pancreatic neuroendocrine tumor, pancreatic neuroendocrine tumors, well-differentiated neuroendocrine tumor (wdnet), who grade 2

## Abstract

Pancreatic neuroendocrine tumours (PNETs) are malignant processes and are identified as being the rarest of all pancreatic cancers. Here we present the case of a 62-year-old male with a background of chronic lymphocytic leukaemia (CLL) who was investigated over 18 months for progressive hepatic cystic lesions with no known aetiology. Imaging revealed main pancreatic duct dilatation without an identifiable intrinsic or extrinsic cause, coupled with enlarged mesenteric lymph nodes. Endoscopic ultrasound identified multiple cystic liver lesions of different sizes, some reflecting the presence of septations. The calibre of the main pancreatic duct was noted to be dilated in the absence of calculi, raising suspicion for a focal inflammatory process or chronic pancreatitis. Fine needle aspiration was performed from the pancreas, liver and lymph nodes; samples were sent for cytological analysis. This revealed the presence of a well-differentiated neuroendocrine tumour, WHO grade 2, across all three sites. The neuroendocrine multidisciplinary team (MDT) classified the tumor as stage T1N1M1a and recommended treatment with somatostatin analogue with palliative intent. This case reflects the diagnostic challenges of atypical PNET presentations and emphasises the crucial role of endoscopic ultrasound with tissue sampling in establishing a definitive diagnosis.

## Introduction

Neuroendocrine neoplasms (NENs) are rare tumours arising from neuroendocrine cells, with an incidence of approximately 8.8 per 100,000 in England [1]. Gastro-entero-pancreatic NENs (GEP-NENs) represent 55-70% of all NENs, with pancreatic neuroendocrine tumours (PNETs) accounting for 12-20% of GEP-NENs and only 1-2% of all pancreatic cancers [2,3].

PNETs are classified as functional (F-PNETs, 30%) when associated with hormone hypersecretion syndromes, or nonfunctional (NF-PNETs, 70%) [4]. NF-PNETs often present with nonspecific symptoms such as abdominal pain, jaundice, or weight loss, or are detected incidentally [5].

We were presented with the diagnostic challenge of an atypical presentation of PNET demonstrated as multiple hepatic cysts, mimicking benign disease on radiological scans. The interval increase in liver lesions raised suspicion for malignancy. Upon review of the literature, it was noted that only 5.3% of patients with hepatic metastasis secondary to PNET present as hepatic cysts on imaging [1].

This reiterates the diagnostic challenges and the utility of further imaging modalities, including endoscopic ultrasound (EUS) and histopathological analysis.

## Case presentation

A 62-year-old male in remission for chronic lymphocytic leukaemia (CLL) had a surveillance CT of the thorax, abdomen and pelvis. This showed a new adrenal nodule. He was followed up with a CT of the adrenals; this CT showed slight thickening/hyperplasia of the left adrenal gland and also incidentally showed some benign-appearing hepatic cysts. A repeat CT adrenal scan after 12 months ruled out an adrenal nodule; however, it noted multiple new hepatic infiltrates which were not evident on the scan from the previous year (Figure 1).

**Figure 1:**
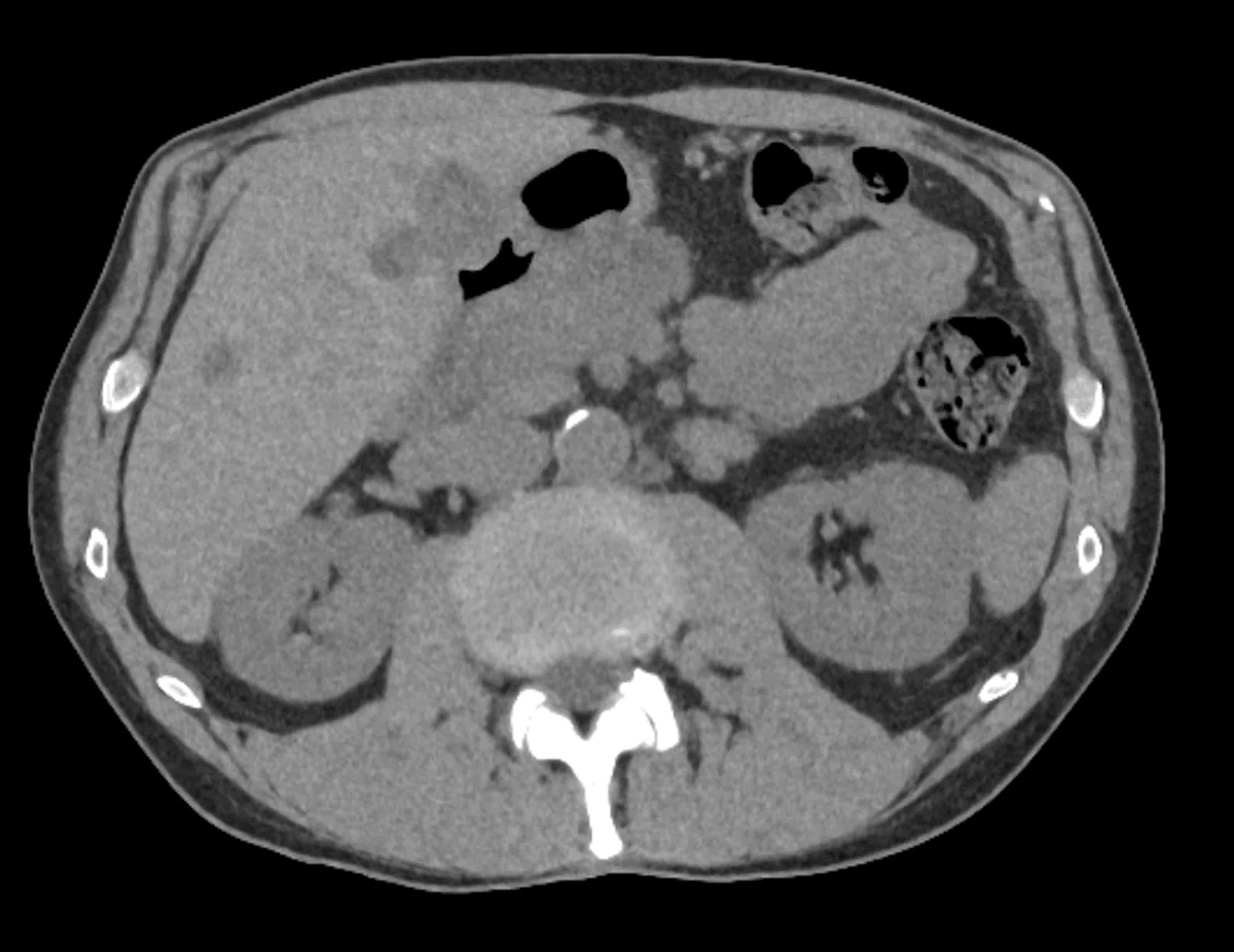
Contrast enhanced CT of the adrenal glands demonstrating multiple newly developed focal hepatic lesions, not present on prior imaging.

At that time, the patient was completely asymptomatic and his routine bloods were unremarkable. Two months after the CT adrenal scan, a PET-CT was performed. This revealed peri-duodenal lymphadenopathy, pancreatic duct enlargement (Figure 2) and bilobar hypoattenuating hepatic lesions without fluorodeoxyglucose (FDG) uptake (Figure 3). A subsequent magnetic resonance cholangiopancreatography (MRCP) was performed after this PET-CT, which showed main pancreatic duct (MPD) dilatation without an intrinsic obstructing lesion (Figure 4). The differentials for these hepatic cysts included mucinous neoplasm, necrotic lesions or benign hepatic cysts. At month three, an ultrasound-guided liver biopsy was arranged; however, this was unsuccessful, yielding no aspirate. Following this, a repeat liver MRI was performed, confirming benign-appearing cystic lesions; however, their rapid development over a four-month period raised concerns for malignancy (the differentials being mucinous tumour or NET metastases) (Figure 5). He was referred to the hepato-pancreato-biliary multidisciplinary team (HPB-MDT), which recommended endoscopic ultrasound (EUS) with tissue sampling; however, the patient declined, as he was asymptomatic and reported being very well during consultations, but he did agree to be followed up with imaging. Within the ninth month of his investigations, a repeat MRI identified over 30 randomly distributed low-density hepatic lesions (Figure 6). Subsequent CT revealed gross MPD dilatation extending to the ampulla, with hepatic lesions substantially increased in size (Figure 7). Two large enhancing lymph nodes were identified on CT pancreas, lateral to the superior mesenteric vein (Figure 8). He was re-discussed in the HPB-MDT, which once again recommended endoscopic ultrasound (EUS) with tissue sampling, which he agreed to undergo.

**Figure 2:**
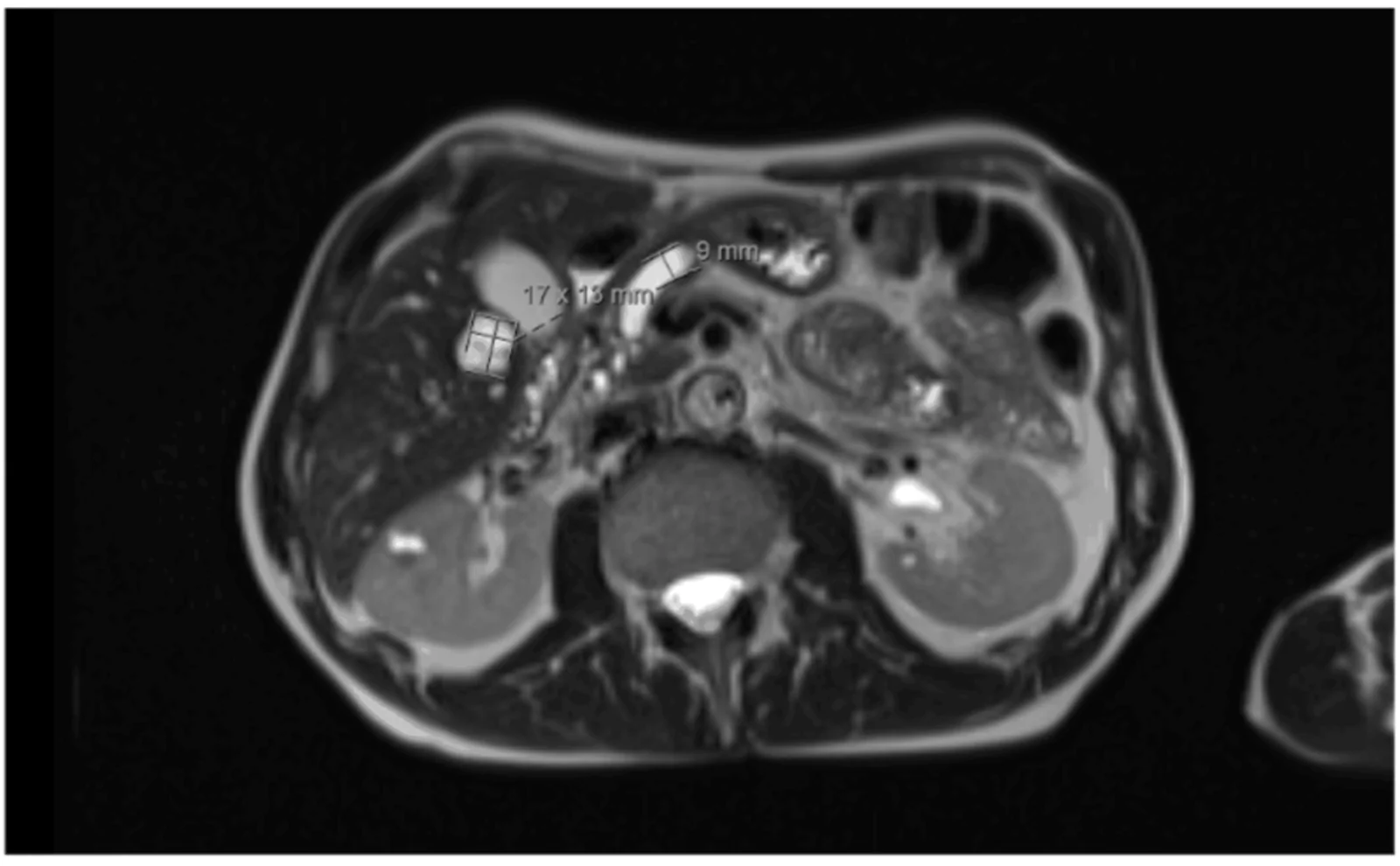
PET CT demonstrating periduodenal lymphadenopathy and dilation of the main pancreatic duct.

**Figure 3:**
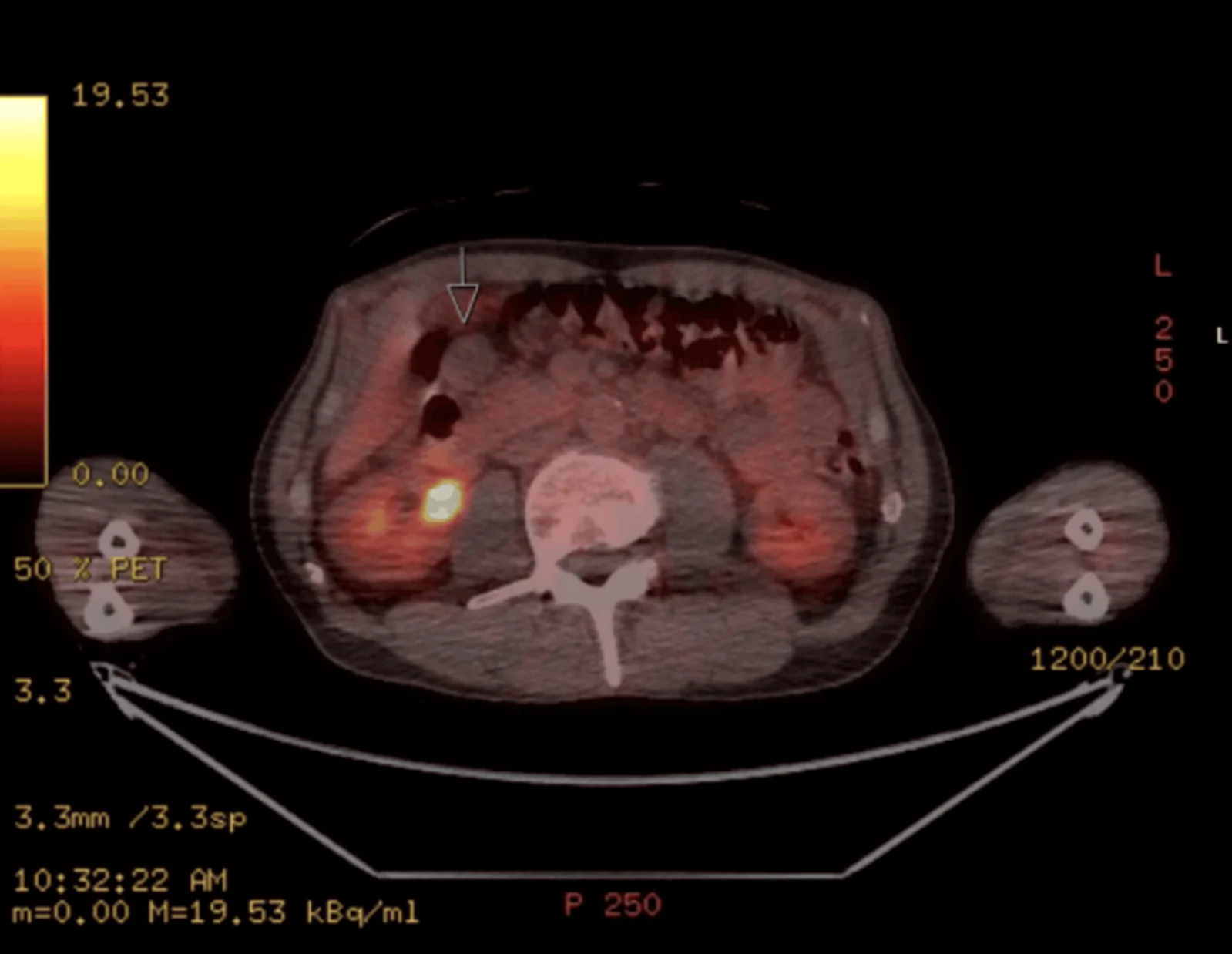
PET-CT demonstrating bilobar hypoattenuating hepatic lesions without FDG uptake. FDG: Fluorodeoxyglucose

**Figure 4:**
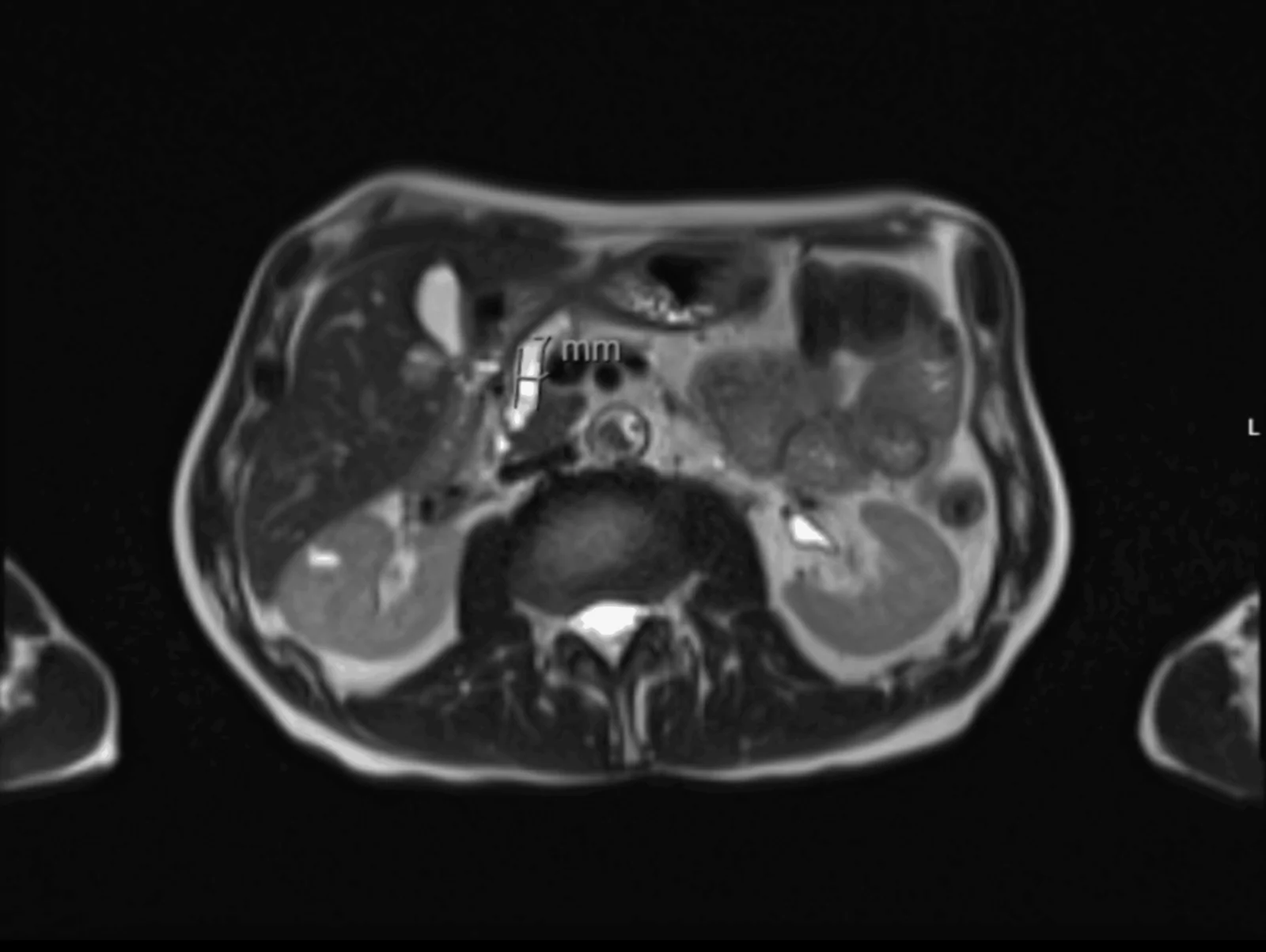
MRCP image demonstrating MPD dilatation in the absence of an intrinsic obstructing lesion. MRCP: Magnetic resonance cholangiopancreatography; MPD: Main pancreatic duct.

**Figure 5:**
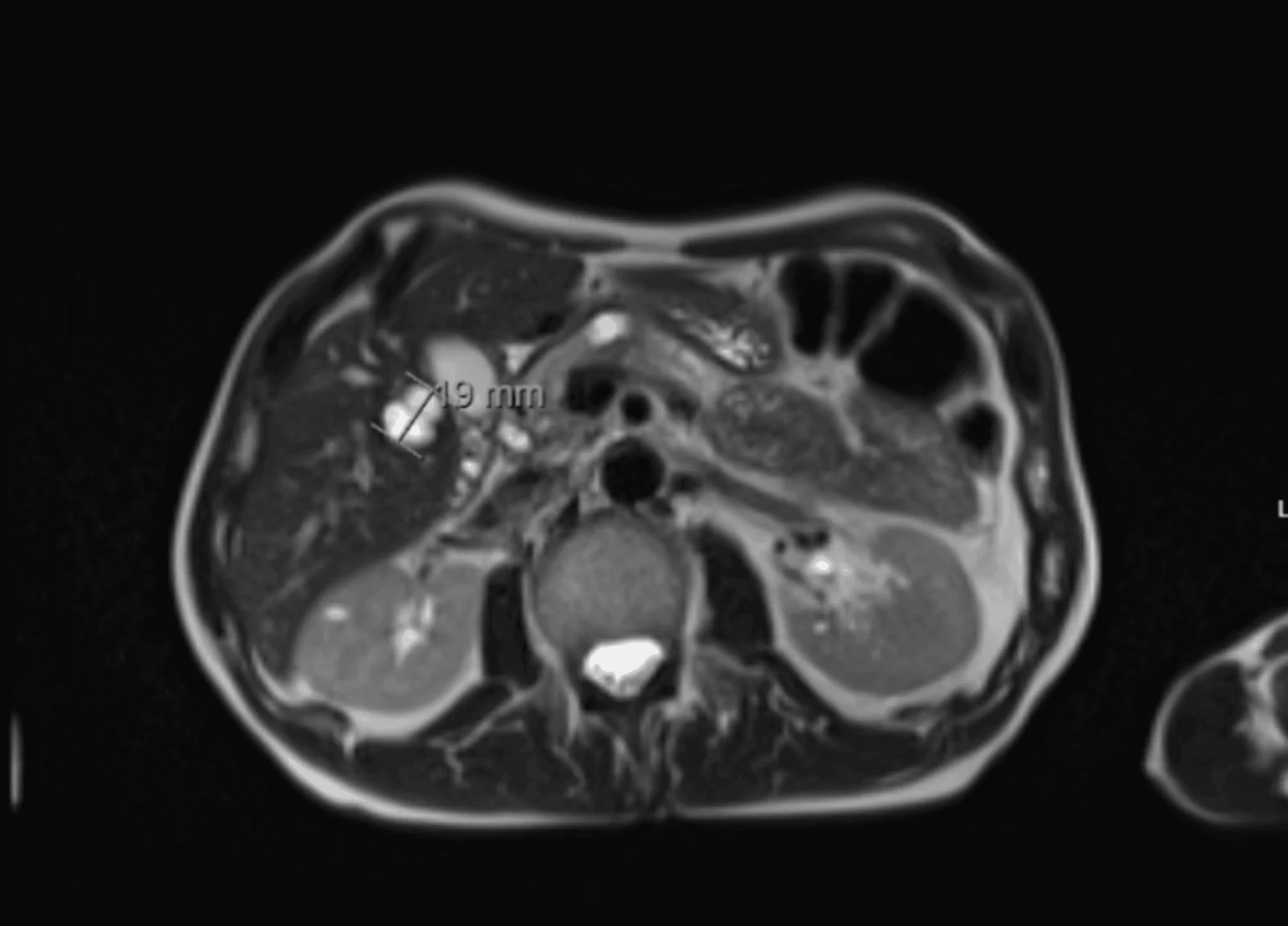
MRI demonstrated benign appearing cystic lesions. However, their rapid interval development raised concerns for possible malignancy.

**Figure 6:**
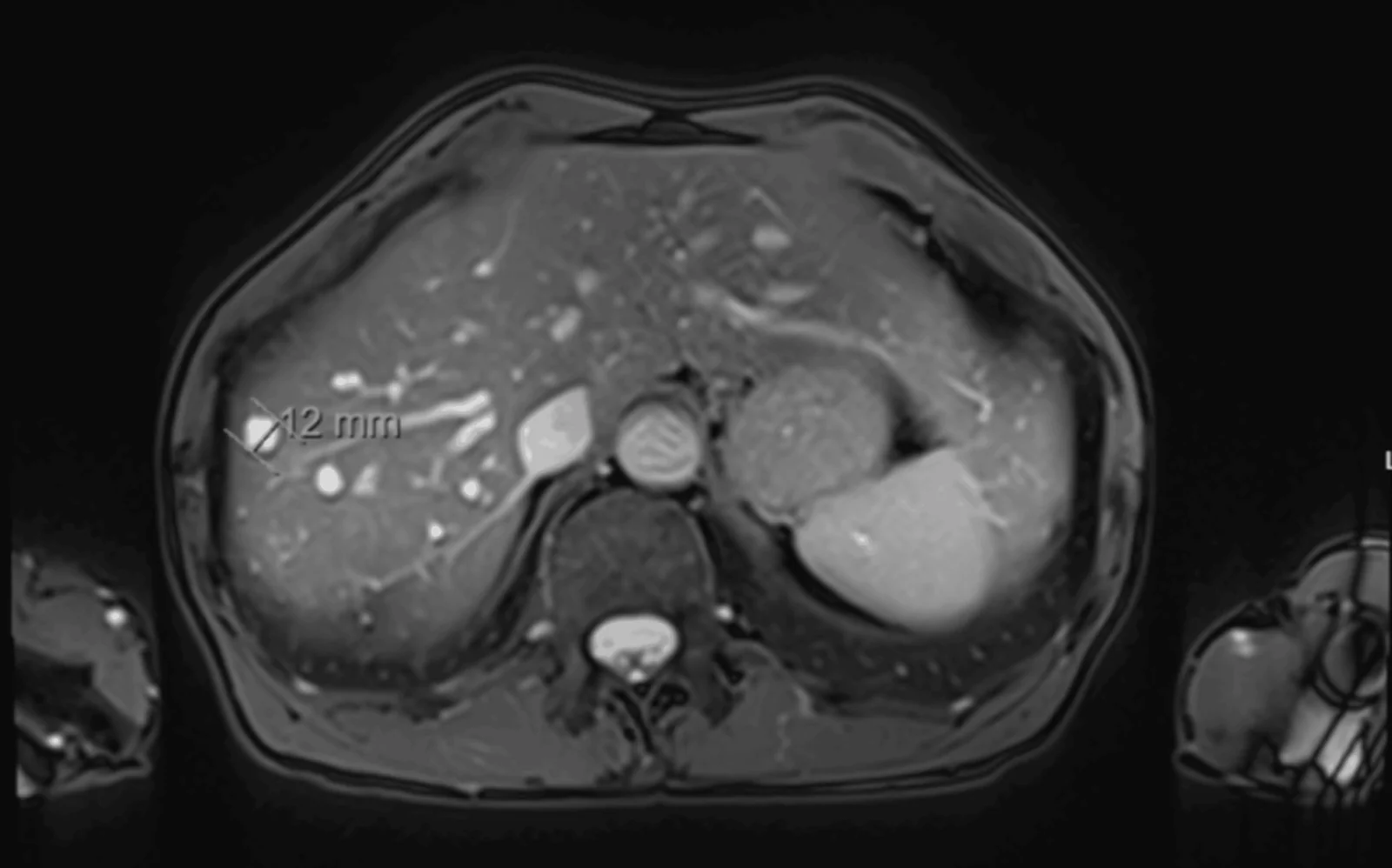
Repeat MRI identified multiple randomly distributed hepatic lesions exhibiting low signal intensity.

**Figure 7:**
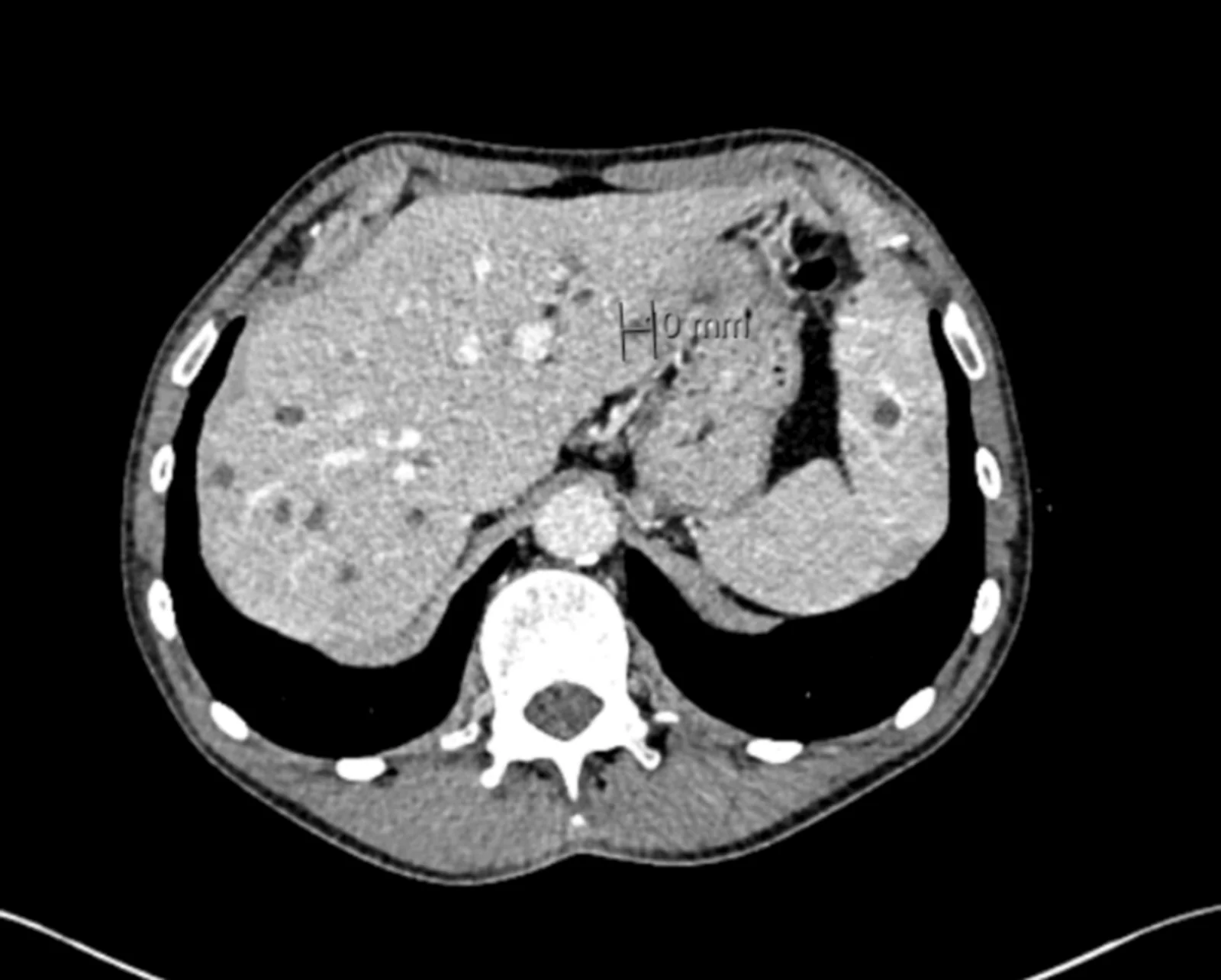
CT of the pancreas revealed marked MPD dilatation extending to the ampulla with substantial interval enlargement of the hepatic lesions. MPD: Main pancreatic duct.

**Figure 8:**
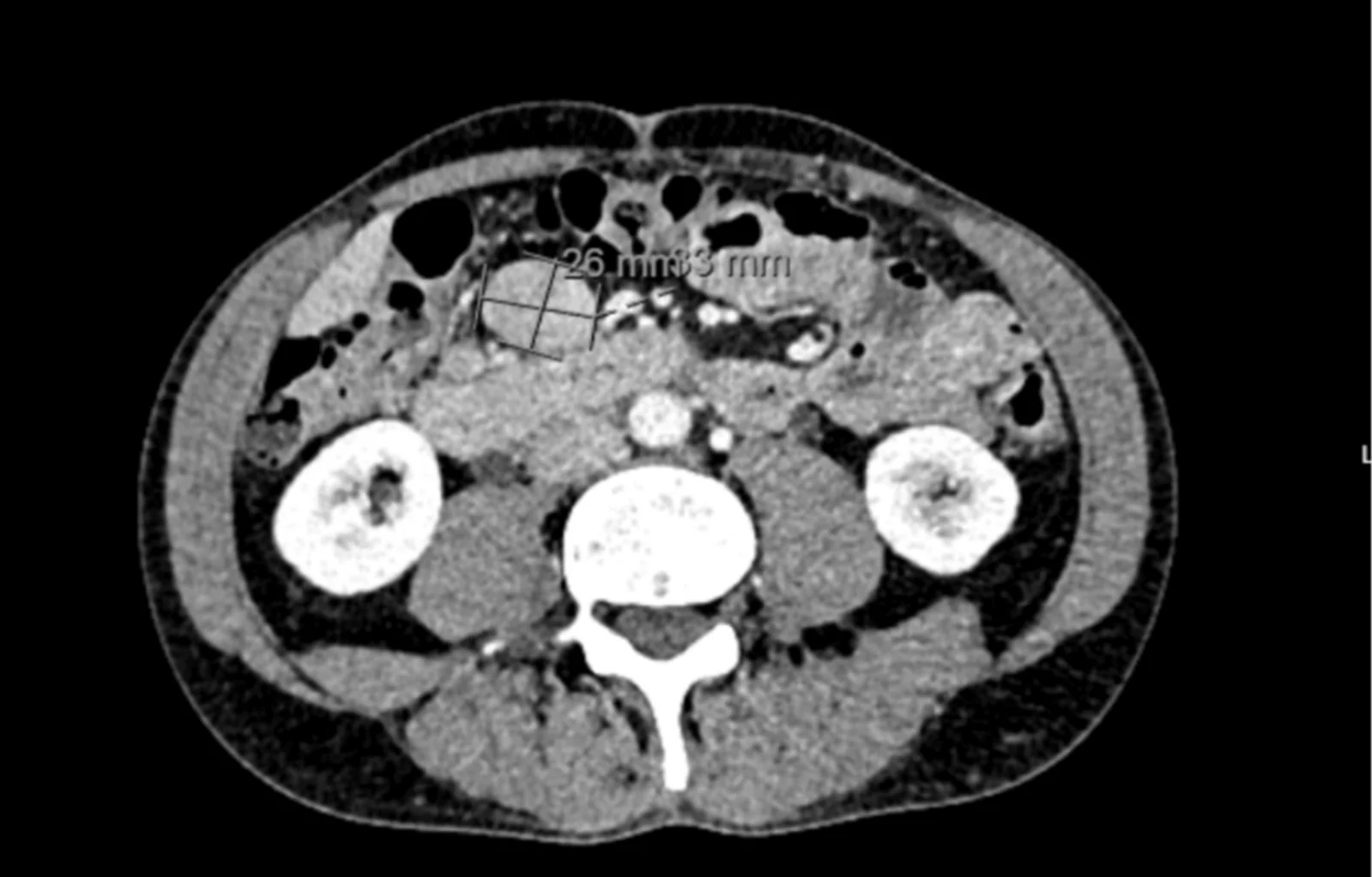
CT pancreas: two large enhancing lymph nodes were identified lateral to the superior mesenteric vein.

Endoscopic ultrasound findings

Pancreas

EUS revealed MPD dilatation within the body and neck of the pancreas with tortuous course and hyperechoic margins. Pancreatic body and tail appeared atrophic, while the head demonstrated generalised hypoechogenicity. MPD caliber variation was observed without complete obstruction (Figures 9-11), suggesting a focal inflammatory process or chronic pancreatitis. Fine needle aspiration (FNA) was obtained from the pancreatic head.

**Figure 9:**
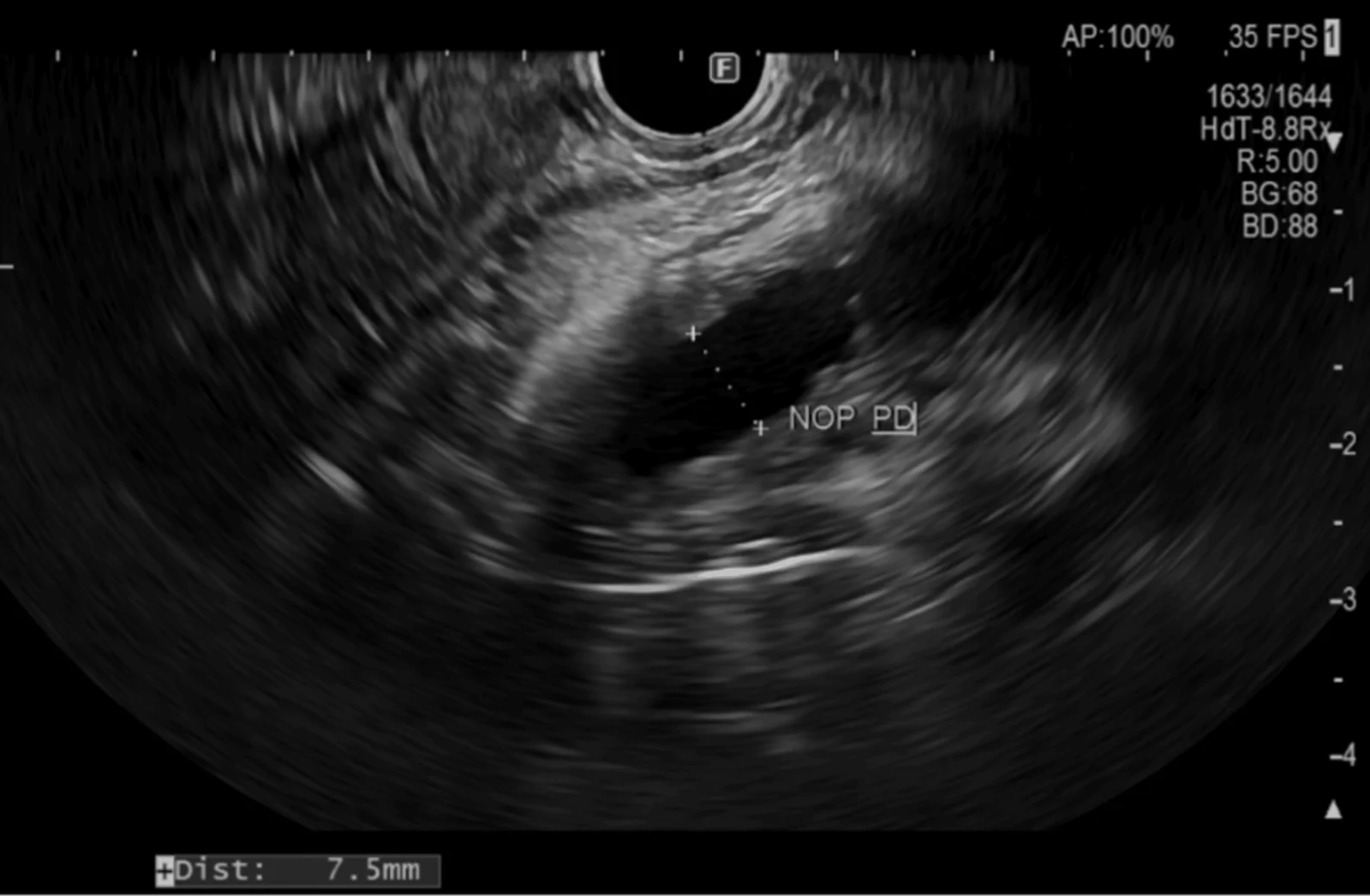
EUS image of the neck of pancreas demonstrating variation in the calibre of the MPD without evidence of ductal obstruction. EUS: Endoscopic ultrasound; MPD: Main pancreatic duct.

**Figure 10:**
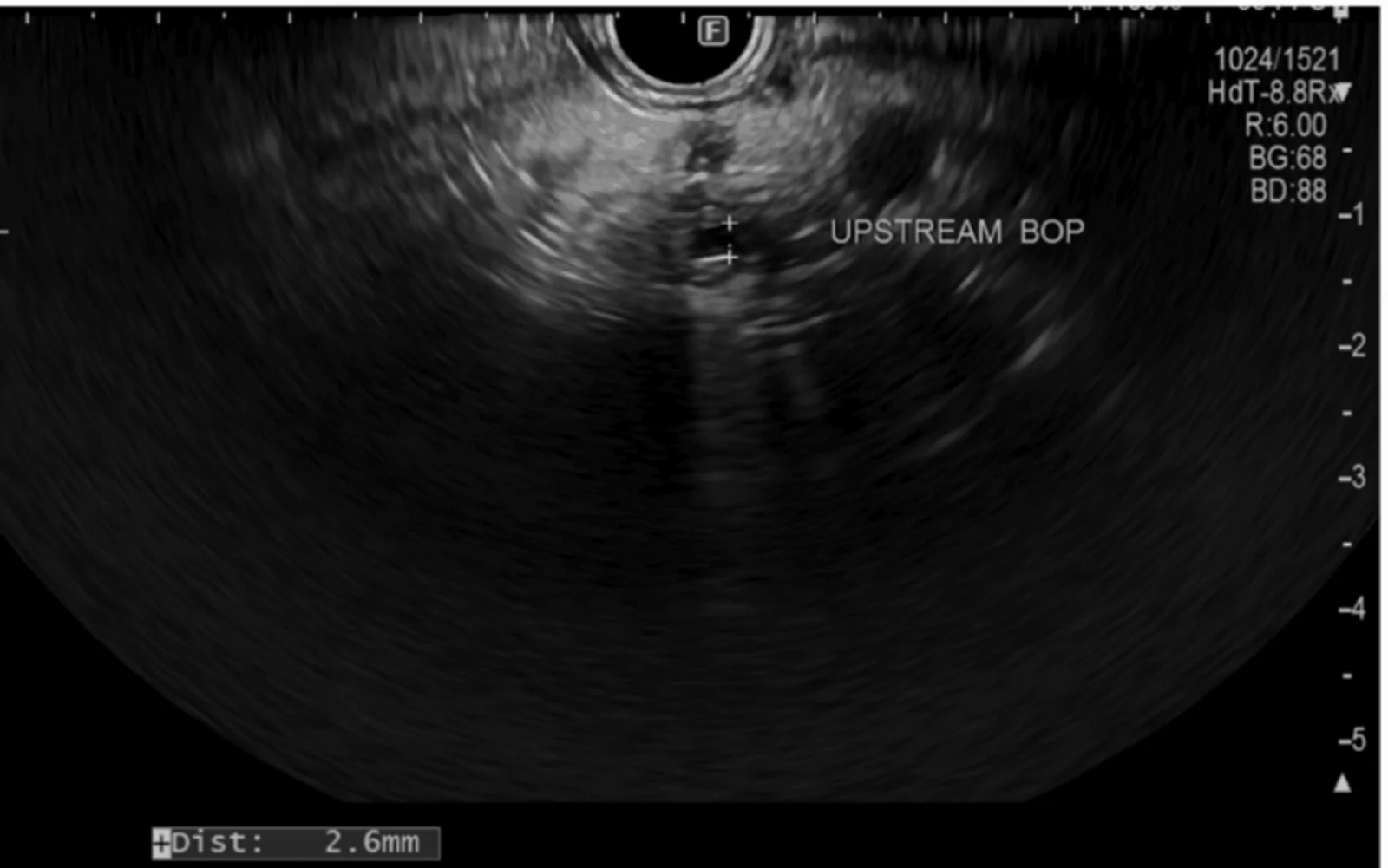
EUS image of the body of pancreas. EUS: Endoscopic ultrasound

**Figure 11:**
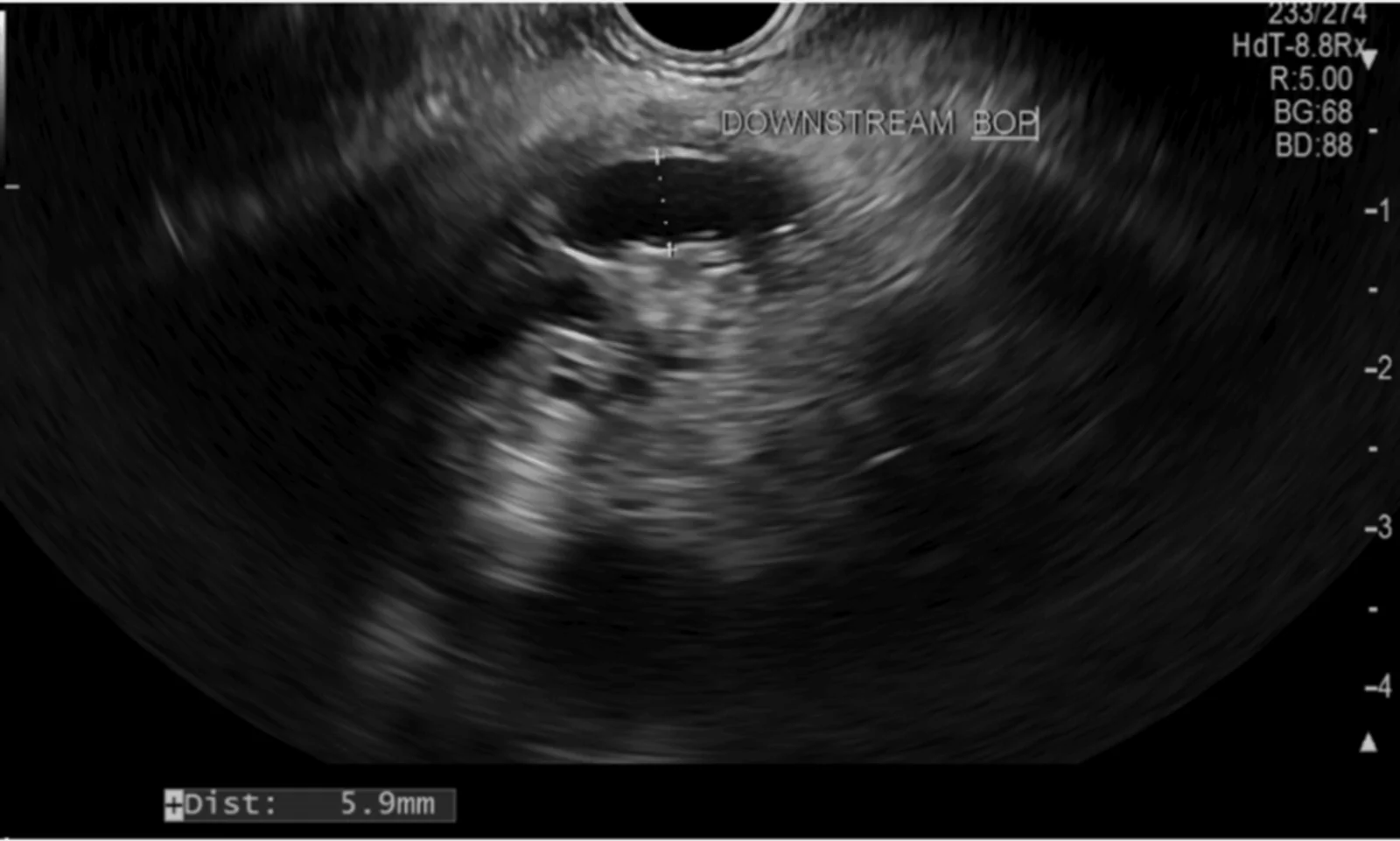
EUS image of the body of pancreas with MPD dilatation. EUS: Endoscopic ultrasound; MPD: Main pancreatic duct.

Liver

EUS demonstrated multiple cystic lesions of varying sizes within the liver, some containing internal septations. FNA yielded straw-coloured fluid submitted for cytological analysis and cultures (Figure 12).

**Figure 12:**
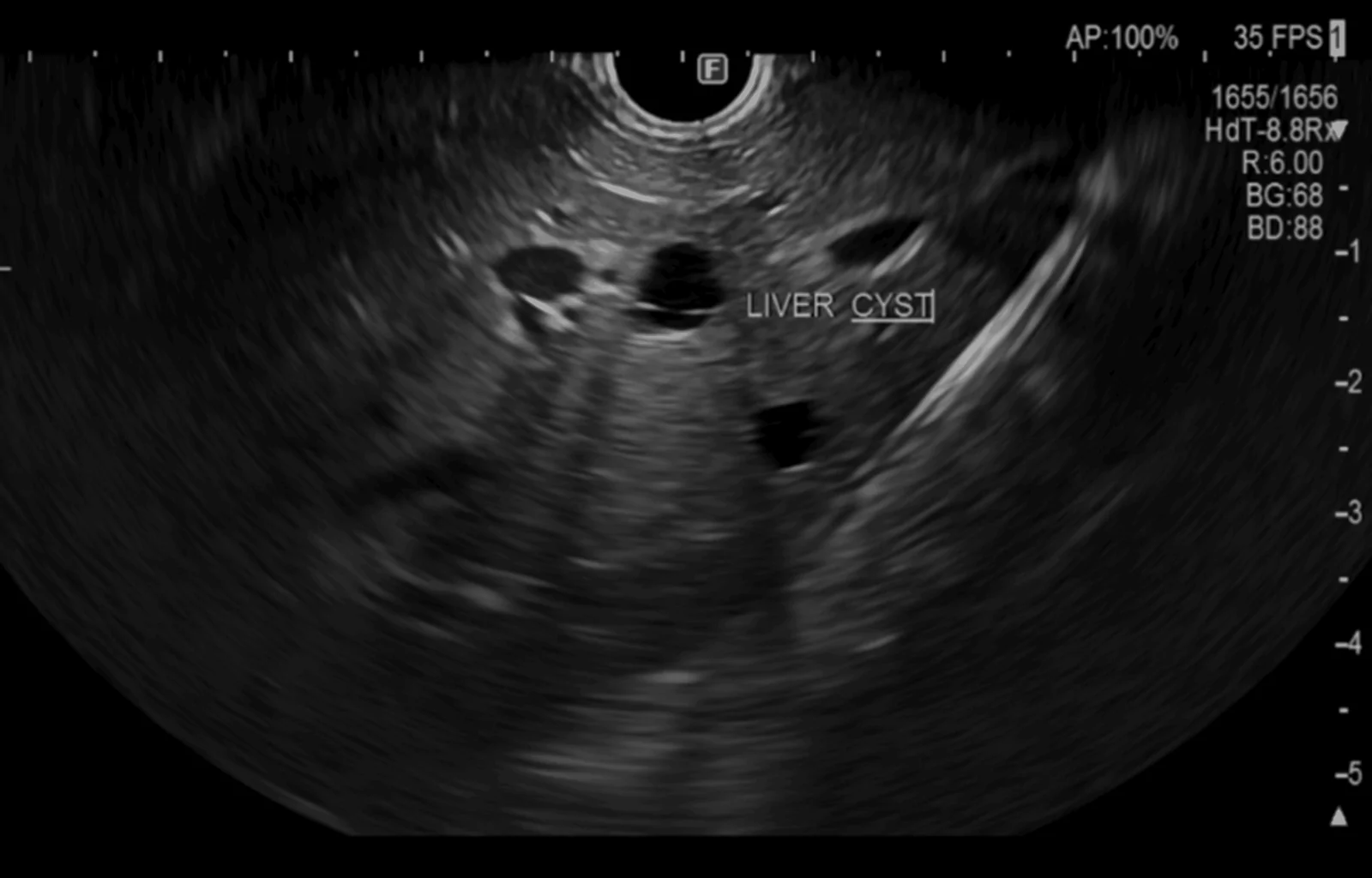
EUS image of the liver - Multiple cystic lesions of varying sizes with internal septations. EUS: Endoscopic ultrasound

Lymph Nodes

Large peripancreatic lymph nodes measuring 25-30 mm were sampled. Cytological examination of all three sites revealed monomorphous plasmacytoid cells consistent with well-differentiated neuroendocrine tumour (WDNET), WHO grade 2. Pancreatic specimens were negative for CD138, insulin, glucagon and calcitonin.

Samples from all three sites and their ranges were evaluated and reported together due to their similar morphologies. Samples were prepared in liquid-based cytospin slides stained with Papanicolaou (PAP) and a formalin-fixed paraffin-embedded (FFPE) clot section stained with hematoxylin and eosin (H&E). Histopathological analysis confirmed a well-differentiated neuroendocrine tumour, intermediate grade (ENETS G2), with immunohistochemistry applied. Histopathological analysis confirmed a well-differentiated, intermediate-grade neuroendocrine tumour (ENETS G2), with immunohistochemistry showing a Ki-67 proliferation index of 8% (Figure 13). The clinical stage was determined as T1N1M1a.

**Figure 13:**
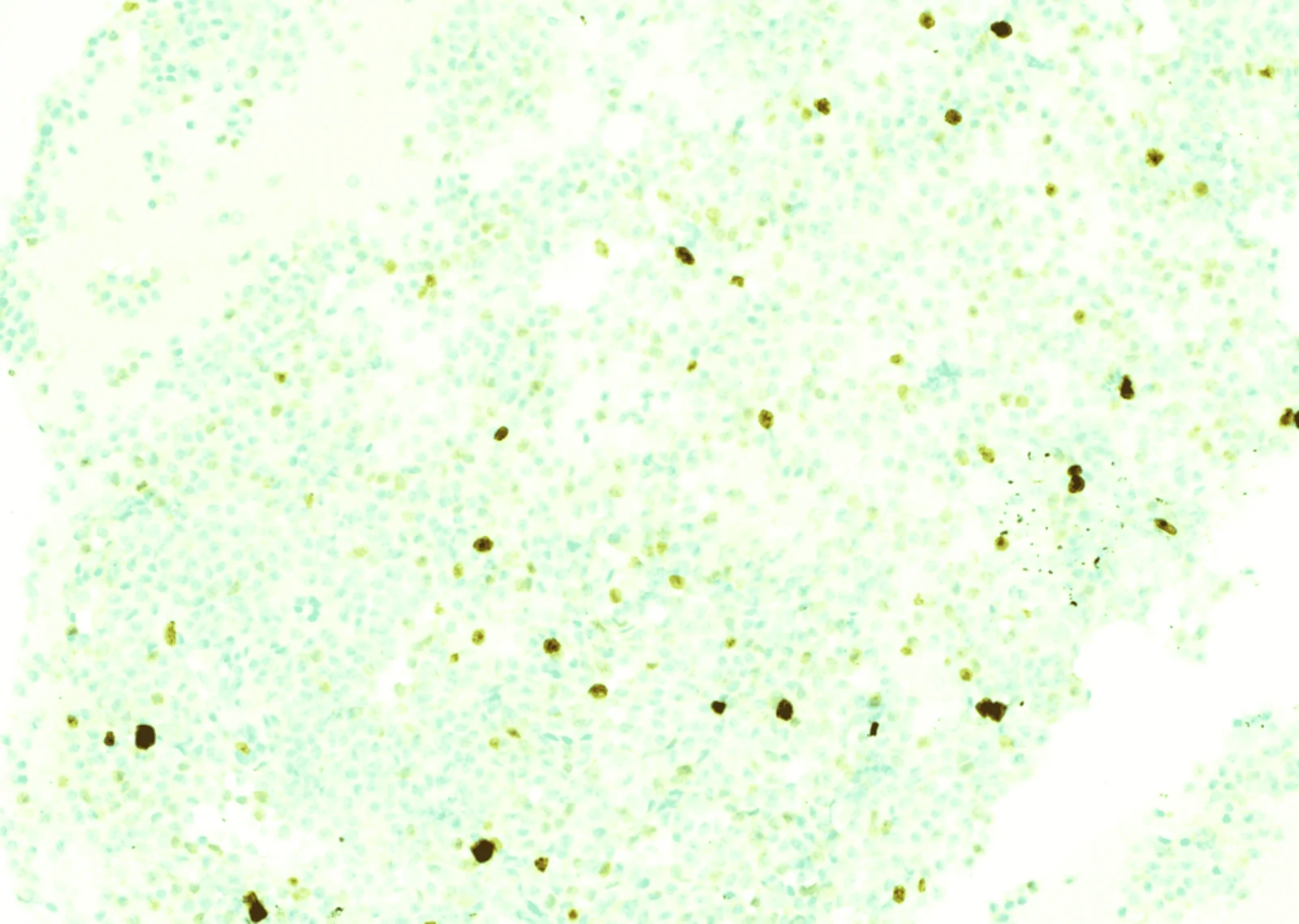
Ki-67 (MIB-1) immunohistochemistry stain applied, noting proliferation of approximately 8% (20x) histology.

The Ga-68 DOTATOC PET/CT scan showed a somatostatin receptor-positive pancreatic head lesion (Figure 14) and adjacent nodal metastases compatible with the biopsy-proven NET.

**Figure 14:**
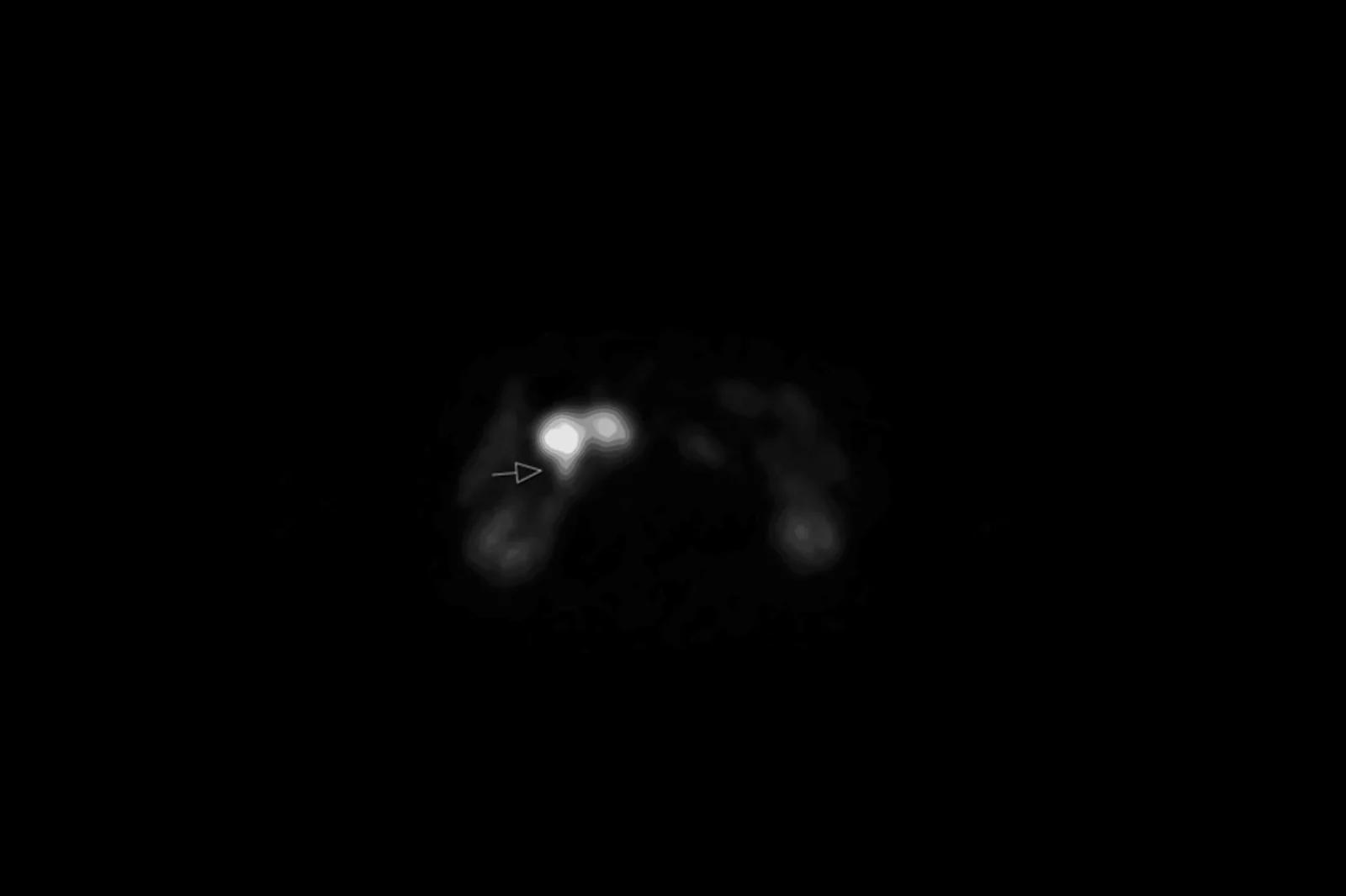
Ga-68 DOTATOC PET/CT (30/10/25) demonstrating a somatostatin receptor-avid lesion in the pancreatic head.

Treatment and assessment of response

The patient was commenced on somatostatin analogues (4-weekly lanreotide) and ongoing follow-up with the Oncology team and radiological surveillance to assess response to treatment.

The above describes a case spanning over 18 months, from initial adrenal node assessment, subsequent incidental finding of hepatic lesions, the monitoring of this on multiple imaging modalities, their rapid multiplication and finally the utilisation of EUS for biopsies. This over one-and-a-half-year process allowed for diagnosis and therefore treatment facilitated by EUS access.

## Discussion

PNETs are classified according to WHO criteria as well-differentiated neuroendocrine tumours (G1, G2, G3) or poorly differentiated neuroendocrine carcinomas [6]. Diagnosis relies on imaging and histopathological confirmation. While CT is the most common initial imaging modality with sensitivity of 63-82% for tumours >2 cm [7], EUS demonstrates superior sensitivity, particularly for lesions <2 cm [8]. EUS allows tissue acquisition through FNA, evaluation of lymph nodes and vascular invasion, and can detect small duodenal gastrinomas and insulinomas that are not visible on other imaging [9].

Most PNETs express somatostatin receptors and demonstrate uptake on somatostatin receptor scintigraphy (Octreoscan), with sensitivity of 75-100% [10]. However, PET scanning has limited utility for well-differentiated PNETs due to their low metabolic activity.

Surgery remains the only curative treatment for PNETs. Pancreatic resection is generally recommended for symptomatic, functional, or tumours ≥2 cm [11]. For unresectable disease, somatostatin analogues constitute first-line therapy for somatostatin receptor-positive tumours, with everolimus and sunitinib as important alternatives [12].

Prognosis depends on tumour grade and stage. Five-year survival rates are 80% for G1, 67% for G2, and 13% for G3 tumours [13]. Localised disease demonstrates 95% five-year survival, whilst distant metastases carry 27% five-year survival [14, 15].

This case illustrates several diagnostic challenges. The cystic appearance of hepatic metastases was atypical, with the differentials being benign disease or mucinous metastasis, in the absence of an identifiable pancreatic mass. The patient's CLL history further complicated interpretation. Ultimately, EUS with FNA of multiple sites proved instrumental in establishing the diagnosis.

## Conclusions

This case demonstrates the diagnostic complexity of PNETs with atypical presentation. The diagnostic journey underscores the importance of maintaining clinical suspicion for neuroendocrine tumours (NETs) in patients with unexplained pancreatic and hepatic abnormalities. EUS with tissue sampling was critical in confirming well-differentiated NET across all three anatomical sites sampled. This case reinforces that even small primary PNETs can present with extensive metastatic disease and emphasises the value of multidisciplinary collaboration in navigating diagnostic uncertainty. As NEN incidence rises, clinicians must remain vigilant for atypical presentations and the utilisation of diagnostic modalities, including CT, MRI, PET-CT and EUS, as early diagnosis and appropriate staging are crucial for optimising treatment strategies and patient outcomes.
